# Author Correction: CryoET reveals actin filaments within platelet microtubules

**DOI:** 10.1038/s41467-024-54642-y

**Published:** 2024-11-28

**Authors:** Chisato Tsuji, Marston Bradshaw, Megan F. Allen, Molly L. Jackson, Judith Mantell, Ufuk Borucu, Alastair W. Poole, Paul Verkade, Ingeborg Hers, Danielle M. Paul, Mark P. Dodding

**Affiliations:** 1 School of Biochemistry, Faculty of Health and Life Sciences, Biomedical Sciences Building, University Walk, University of Bristol, BS8 1TD Bristol, UK; 2 School of Physiology, Pharmacology and Neuroscience, Faculty of Health and Life Sciences, Biomedical Sciences Building, University Walk, University of Bristol, BS8 1TD Bristol, UK; 3 GW4 Facility for High-Resolution Electron Cryo-Microscopy, University of Bristol, Bristol, UK

**Keywords:** Actin, Cryoelectron tomography

Correction to: *Nature Communications* 10.1038/s41467-024-50424-8, published online 16 July 2024

In the Acknowledgements section, the BHF funder grant number ‘PG/21/10760’ was duplicated and should have read ‘FS/19/53/34887’. The original article has been corrected.

