# Supplementary material for: CryoET reveals actin filaments within platelet microtubules
Source: Nat Commun. 2024 Jul 16;15:5967. doi: 10.1038/s41467-024-50424-8 (PMC11252303; doi:10.1038/s41467-024-50424-8)
Supplement: Supplementary file 1 — Supplementary Figs. [file 41467_2024_50424_MOESM1_ESM.pdf]

Supplementary Information for:

CryoET reveals actin filaments within platelet microtubules

Chisato Tsuji[1], Marston Bradshaw[2], Megan F. Allen[2], Molly L. Jackson[2], Judith Mantell[1], Ufuk Borucu[3],

Alastair W. Poole[2], Paul Verkade[1], Ingeborg Hers[2]*, Danielle M. Paul[2]* and Mark P. Dodding[1]*

[1] School of Biochemistry, Faculty of Health and Life Sciences, Biomedical Sciences Building, University Walk, University of Bristol, BS8 1TD.

[2] School of Physiology, Pharmacology and Neuroscience, Faculty of Health and Life Sciences, Biomedical Sciences Building, University Walk, University of Bristol, BS8 1TD.

[3] GW4 Facility for High-Resolution Electron Cryo-Microscopy, University of Bristol.

* Correspondence to mark.dodding@bristol.ac.uk; danielle.paul@bristol.ac.uk; i.hers@bristol.ac.uk

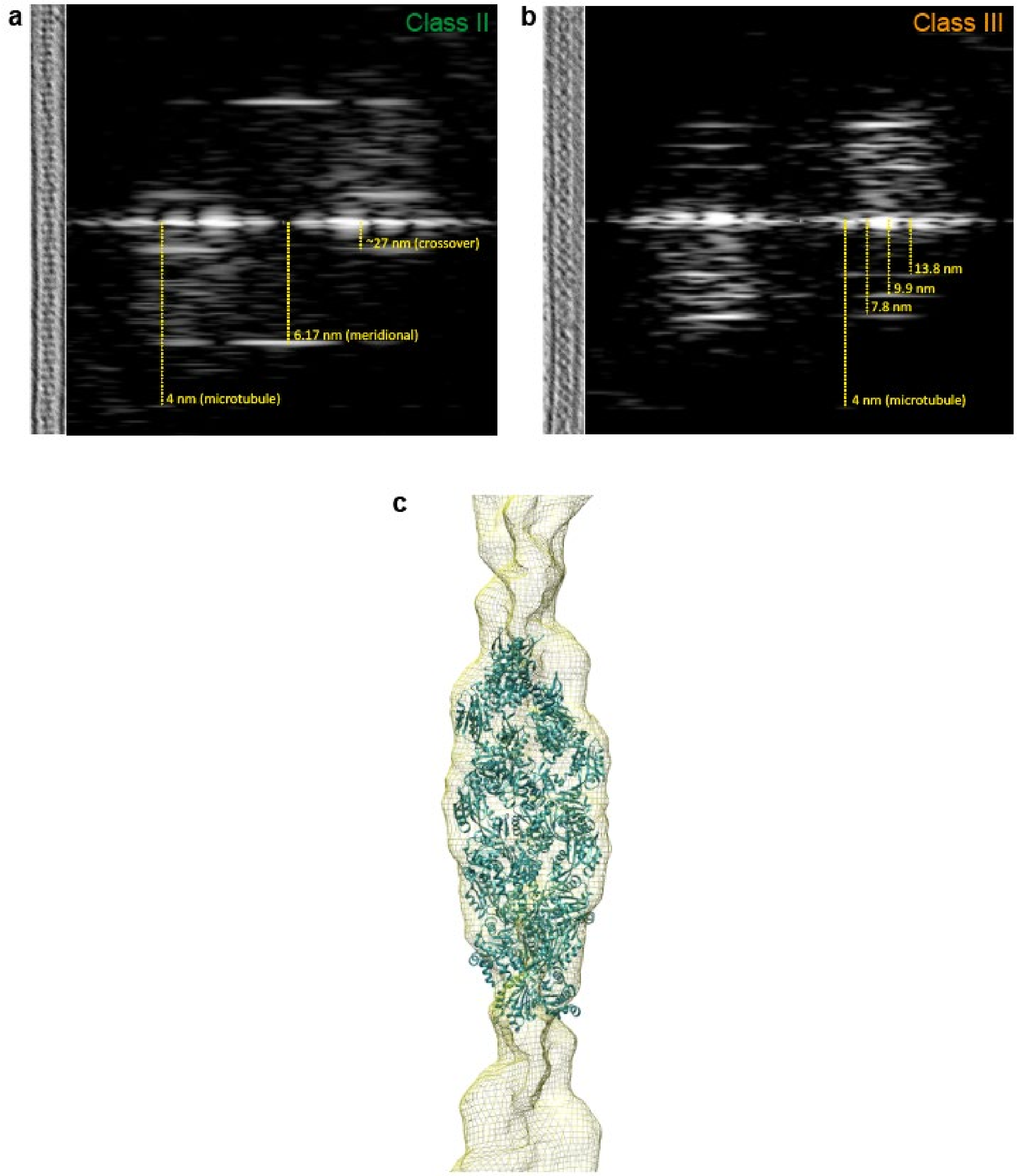

Supplementary Fig. 1. Characterisation of lumenal filaments. (a,b) Power spectra of Class II and Class III filaments annotated with real space distances. **(c)** Docking of cofilin-actin structure PDB:8OH4 into map shown in Fig. 1H.

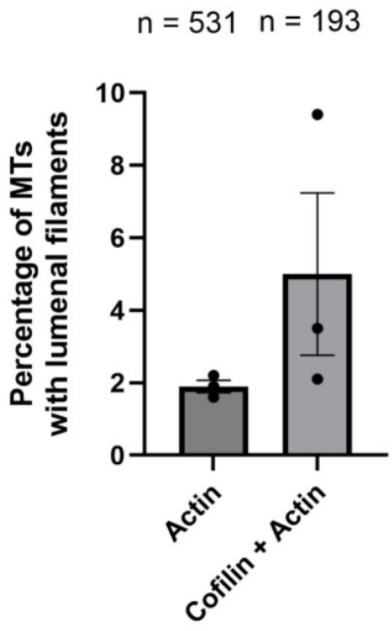

Supplementary Fig. 2. Quantification of *in vitro* reconstitution data. Three polymerisation experiments were performed for each condition (tubulin and actin, or tubulin and actin plus cofilin) and microtubules (n = 531 and n=193 respectively, in total) were counted. Error bars show S.E.M. There was no significant difference using a 2-tailed t-test (p=0.1).

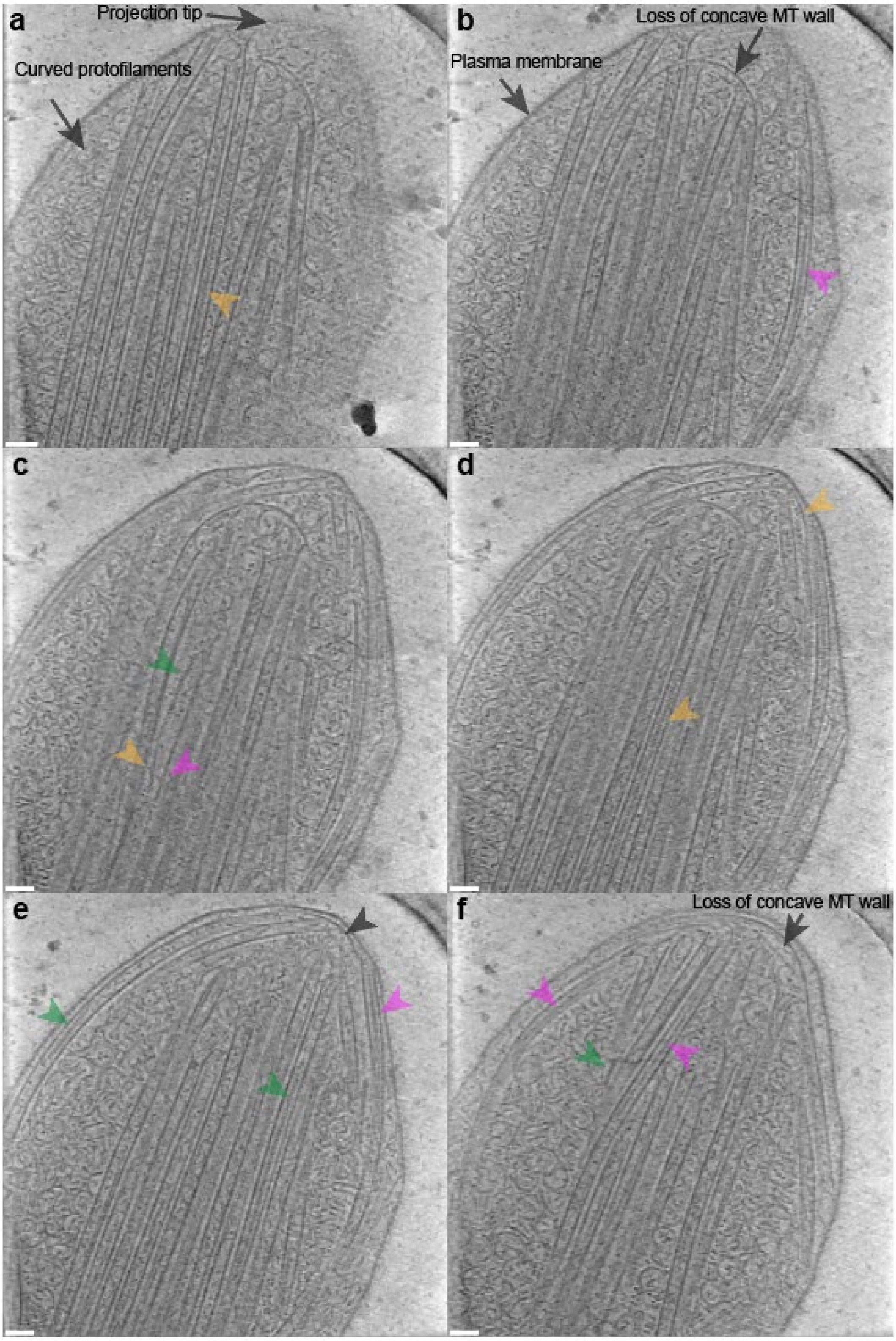

Supplementary Fig. 3. Microtubules are under structural and mechanical stress when they are extruded from HAP1 cells. Tomogram slices showing a series of images along the Z-axis of the tip of a projection **(a-f).** The three filament classes are highlighted by coloured arrows (magenta – Class I, green Class II, orange, Class III). A video showing this Z-series is provided in Supplementary Movie 1. Scale bar = 50 nm.

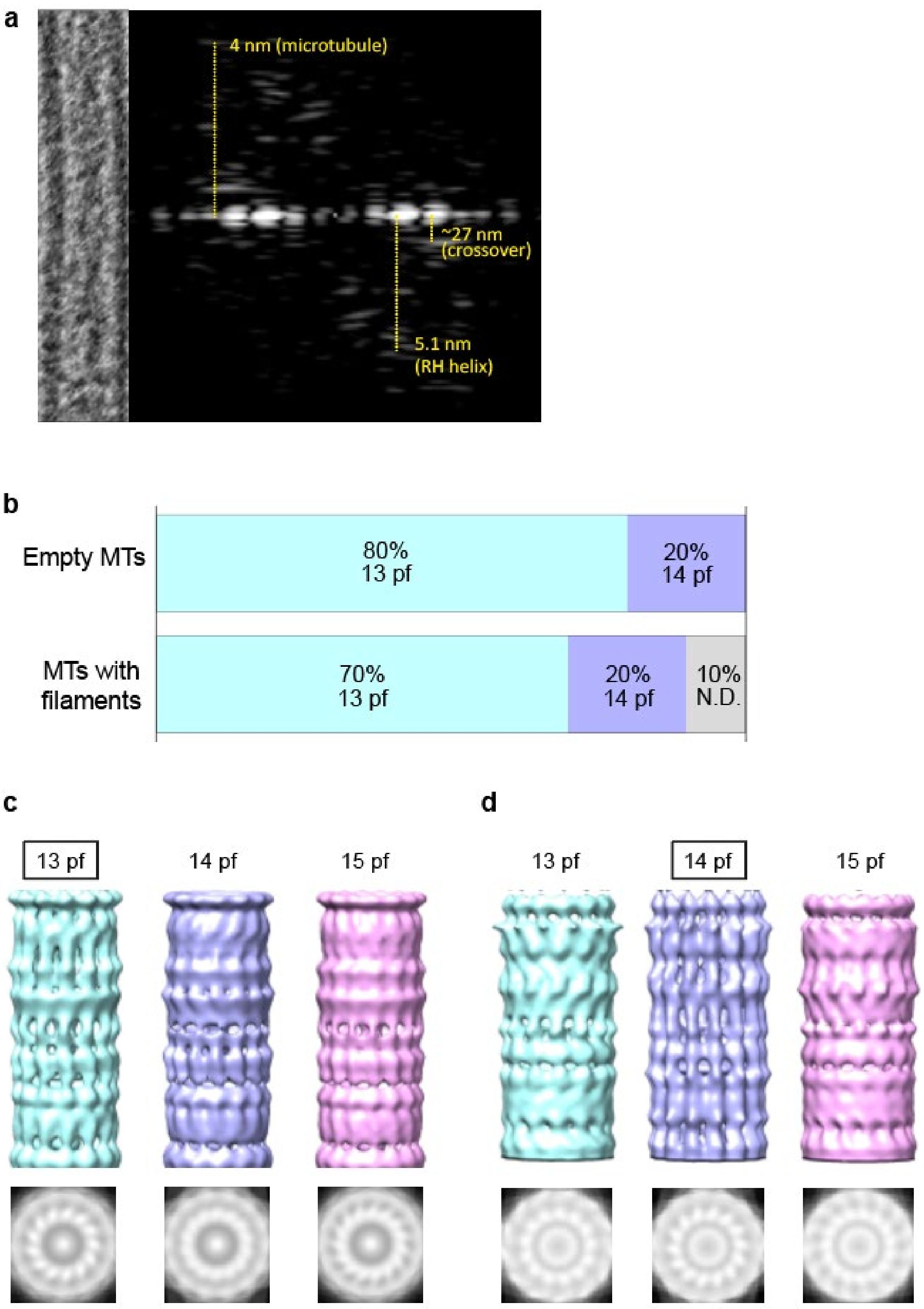

Supplementary Fig. 4 Characterisation of microtubules and lumenal filaments in platelets. (a) Power spectrum of a lumenal Class I filaments in platelet microtubules annotated with real space distances. **(b)** Quantification of the protofilament number for 10 microtubules in platelets that do and do not contain filaments. **(c, d)** Representative examples of cross sections of rotationally averaged segments of microtubules and their associated 3D models viewed on UCSF Chimera with C13, C14 or C15 symmetries. The example in **(c)** was identified as a 13 protofilament microtubule whereas **(d)** was identified as a 14 protofilament microtubule.
