# Supplementary material for: CryoET reveals actin filaments within platelet microtubules
Source: Nat Commun. 2024 Jul 16;15:5967. doi: 10.1038/s41467-024-50424-8 (PMC11252303; doi:10.1038/s41467-024-50424-8)
Supplement: Supplementary file 3 — Description of Additional Supplementary Files [file 41467_2024_50424_MOESM3_ESM.pdf]

Supplementary Movie 1.
CryoET analysis of tips of HAP1 cell projections. Image series through the tomogram depicted in Supplementary Fig. 3 showing a projection tip. Scale bar is 50 nm.

Supplementary Movie 2.
CryoET analysis of platelet microtubules. Image series through the tomogram depicted in Fig. 3B showing microtubules in a FIB-milled platelet. Scale bar is 50 nm.
